# Hemodynamics of Proximal Coronary Lesions in Patients Undergoing Transcatheter Aortic Valve Implantation: Patient-Specific In Silico Study

**DOI:** 10.3390/bioengineering12040339

**Published:** 2025-03-26

**Authors:** Yahia Bellouche, Sirine Abdelli, Sinda Hannachi, Clement Benic, Florent Le Ven, Romain Didier

**Affiliations:** 1 Cardiology Department, Brest University Hospital (CHRU Brest), 29200 Brest, France; sirine.abdelli@chu-brest.fr (S.A.); sinda.hannachi@chu-brest.fr (S.H.); clement.benic@chu-brest.fr (C.B.); florent.leven@chu-brest.fr (F.L.V.); romain.didier@chu-brest.fr (R.D.); 2 Medicine Faculty, Western Brittany University (UBO), 29200 Brest, France; 3 Western Brittany Thrombosis Study Group, Inserm UMR 1304 (GETBO), Western Brittany University Brest, 29200 Brest, France

**Keywords:** aortic stenosis (AS), coronary artery disease (CAD), transcatheter aortic valve implantation (TAVI), computational fluid dynamics (CFD), fractional flow reserve (FFR), instantaneous wave-free ratio (iFR), wall shear stress (WSS)

## Abstract

Aortic stenosis (AS) frequently coexists with coronary artery disease (CAD), complicating revascularization decisions. The use of coronary physiology indices, such as the fractional flow reserve (FFR), instantaneous wave-free ratio (iFR), and coronary flow reserve (CFR), in AS patients remains debated, particularly after transcatheter aortic valve implantation (TAVI). In this study, we employ computational fluid dynamics (CFD) to evaluate coronary hemodynamics and assess changes in the wall shear stress (WSS) before and after TAVI. Our analysis demonstrates strong agreement between CFD-derived and invasive FFR measurements, confirming CFD’s reliability as a non-invasive tool for coronary physiology assessment. Furthermore, our results show no significant changes in FFR (p=0.92), iFR (p=0.67), or CFR (p=0.34) post-TAVI, suggesting that these indices remain stable following aortic valve intervention. However, a significant reduction in high WSS exposure (59% to 40.8%, p<0.001) and the oscillatory shear index (OSI: 0.32 to 0.21, p<0.001) was observed, indicating improved hemodynamic stability. These findings suggest that coronary physiology indices remain reliable for revascularization guidance post-TAVI and highlight a potential beneficial effect of aortic stenosis treatment on plaque shear stress dynamics. Our study underscores the clinical utility of CFD modeling in CAD management, paving the way for further research into its prognostic implications.

## 1. Introduction

Degenerative calcified aortic stenosis (AS) is a complex pathology that shares the same risk factors with atherosclerosis, such as age, diabetes, hypertension, and hyperlipidemia. The presence of coronary artery disease (CAD) in patients with severe AS is commonly observed, and its prevalence increases with age and the degree of valve calcification. A comprehensive study conducted in Sweden revealed that concomitant coronary artery bypass grafting (CABG) is performed in a substantial proportion of AS patients across different age groups. The rates of CABG were found to be 7.2% in patients aged <50 years, 30.2% in patients aged between 51 and 60 years, 41.2% in patients aged 61 to 70 years, and 51.2% in patients aged >71 years [1], in another work by Thalji et al. and Malmberg et al., the prevalence of CAD in patients with severe AS varied between 15 and 80% and it was found in about 30% of patients undergoing surgical aortic valve replacement [2,3]. Another investigation involving 308 patients with aortic valve calcification and coronary angiography demonstrated a significant association between aortic valve calcification and the presence of significant coronary lesions [4]. Similarly, an analysis of the France-2 TAVI registry, which encompassed 4201 patients without a history of bypass surgery, indicated a 30% prevalence of coronary artery disease, with multi-vessel disease observed in half of these cases. Additionally, the extent of coronary artery disease was found to correlate with an increased cardiovascular risk profile and EuroSCORE logistic operative risk score [5]. These findings emphasize the high prevalence of CAD in patients with severe AS and highlight the need for a thorough evaluation of coronary lesions in this population when determining the optimal treatment strategy. Understanding the extent and severity of coronary artery disease in AS patients can significantly impact risk assessment and management decisions, ultimately improving patient outcomes.

The optimal revascularization (defined as the absence of functionally significant [with a fractional flow reserve (FFR) > 0.80] coronary lesions) strategy for patients with severe AS and concomitant CAD remains a matter of debate. The choice between percutaneous coronary intervention (PCI) and coronary artery bypass grafting (CABG) depends on multiple factors, including lesion complexity, the extent of CAD, and individual patient characteristics. The advent of TAVI has further complicated this decision-making process. TAVI provides a less invasive treatment option for AS, but it brings new challenges in terms of revascularization. The interaction between the aortic valve, coronary arteries, and myocardial perfusion requires a comprehensive understanding to guide treatment decisions effectively and obtain optimal revascularization.

**Figure 1:**
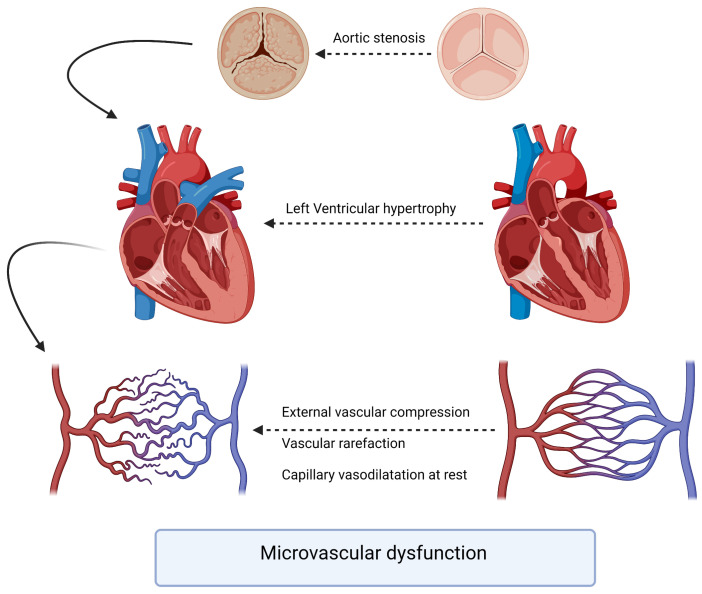
Pathophysiology of microvascular dysfunction in aortic stenosis patients.

The pathophysiology underlying the interaction between aortic stenosis and coronary arteries involves a complex interplay of hemodynamic, structural, and molecular factors. As AS progresses, the left ventricle faces increased afterload due to the narrowing of the aortic valve orifice. The chronic pressure overload leads to left ventricular hypertrophy, which, in turn, affects coronary perfusion. The increased myocardial oxygen demand may outpace the ability of the coronary arteries to supply adequate blood flow (Figure 1). A compromised coronary perfusion can result in myocardial ischemia and impaired cardiac function. To assess the severity and functional significance of coronary artery lesions, physiological indices such as the fractional flow reserve (FFR) and instantaneous wave-free ratio (iFR) have gained prominence. FFR is the gold standard for evaluating the hemodynamic significance of coronary stenoses by measuring the pressure gradient across the lesion during maximal hyperemia. FFR values < 0.81 are typically considered indicative of hemodynamically significant stenoses warranting revascularization [6].

However, in the presence of aortic stenosis, the use of FFR for decision-making becomes more complex. The altered hemodynamics caused by aortic stenosis can affect FFR measurements and potentially lead to underestimation of coronary lesion severity. Resting coronary flow may remain relatively preserved in patients with aortic stenosis, while hyperemic flow may increase after TAVI due to improved left ventricular function [7]. These factors may influence FFR values and necessitate the adjustment of cutoff thresholds to determine the need for revascularization. Similarly, iFR has emerged as a non-hyperemic alternative to FFR for assessing coronary lesions. iFR measures the pressure gradient across a lesion during a specific phase of the cardiac cycle, known as the wave-free period, which occurs naturally without the need for pharmacological hyperemia. iFR values <0.90 are considered indicative of hemodynamically significant stenoses [6]. However, further studies are needed to determine the optimal use of iFR in patients with aortic stenosis and concomitant CAD. Therefore, while coronary physiology indices offer valuable insights for decision-making, their interpretation and cutoff thresholds need to be carefully adjusted in the context of aortic stenosis. This requires comprehensive clinical and hemodynamic evaluations to account for unique hemodynamic conditions in patients with aortic stenosis and coronary artery disease.

In recent years, computational fluid dynamics (CFD) has emerged as a key tool in cardiovascular medicine, providing a sophisticated means to analyze hemodynamic parameters in both physiological and pathological conditions. By integrating patient-specific imaging data with advanced numerical modeling techniques, CFD enables noninvasive assessment of blood flow dynamics, pressure gradients, and wall shear stress (WSS), offering insights that would otherwise necessitate invasive procedures. This capability has been instrumental in advancing the understanding of vascular pathophysiology, refining interventional strategies, and optimizing the design of cardiovascular devices.

**Figure 2:**
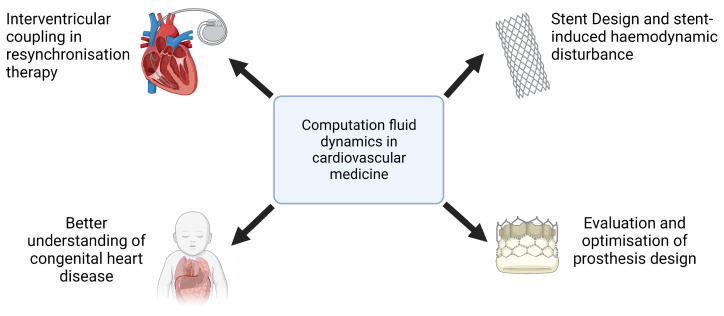
Examples of computational fluid dynamics applications in cardiovascular medicine.

The clinical applications of CFD cover a wide range of cardiovascular conditions (Figure 2). In the assessment of coronary artery disease (CAD), the CFD-derived fractional flow reserve from coronary computed tomography angiography (FFRCT) has demonstrated strong concordance with invasive FFR measurements, providing a valuable tool for the functional evaluation of coronary stenoses and guiding percutaneous coronary intervention (PCI) decisions [8,9]. Similarly, in the treatment of aortic pathologies, CFD has been used to predict post-stent hemodynamics in patients undergoing endovascular aneurysm repair, helping to refine stent graft designs and mitigate flow disturbances that contribute to endoleak formation [10,11]. The role of CFD in valvular heart disease has also been increasingly recognized, particularly in the context of transcatheter aortic valve implantation (TAVI). Studies employing CFD methodologies have characterized postprocedure flow patterns, elucidated pressure recovery phenomena, and quantified alterations in WSS, thus informing the selection of prosthetic valve sizes and implantation strategies [12]. Beyond macrovascular applications, CFD has also been used in the study of microvascular dysfunction, providing mechanistic insights into conditions such as coronary microvascular disease and pulmonary hypertension.

Furthermore, CFD plays a critical role in the development of cardiovascular implants and assistive devices, including the optimization of artificial heart valves, ventricular assist devices, and stents, where numerical simulations help to reduce thrombotic risk and improve device performance [13]. The ability of CFD to replicate patient-specific hemodynamic conditions underscores its potential as a transformative tool in precision medicine, facilitating individualized therapeutic planning and advancing the field of computational cardiology.

This paper seeks to investigate changes in coronary physiological CFD-derived indices (specifically FFR, iFR, and CFR) before and after aortic valve replacement to assess their reliability and safety in guiding revascularization decisions. Furthermore, as a secondary analysis, our objective is to evaluate variations in the regional wall shear stress adjacent to proximal lesions following treatment for aortic stenosis. This analysis aims to elucidate any potential benefits of aortic stenosis treatment on plaque shear stress, a factor associated with ischemic events [14].

## 2. Methods

This retrospective, patient-specific, data-based study aimed to investigate the hemodynamic behavior of coronary lesions in patients with severe aortic stenosis (AS) and concomitant coronary artery disease (CAD). Using computational fluid dynamics (CFD) simulations with the SIMVASCULAR platform, the study focused on evaluating variations in hyperemic indices (FFR, CFR) and non-hyperemic indices (iFR) of coronary proximal lesions as a primary end point and the absence of significant change as a hypothesis, along with the Time Average Wall Shear Stress (TAWSS) and the Oscillatory Shear Index (OSI), before and after aortic valve replacement. Through accurate simulation of blood flow dynamics and analysis of key hemodynamic parameters, we aimed to enhance our understanding of how aortic stenosis affects coronary flow and wall shear stress, and how these dynamics change following aortic valve replacement. Patient-specific imaging data, such as computed tomography (CT) or magnetic resonance imaging (MRI), were used to create 3D models of the aortic valve, coronary arteries, and surrounding vasculature. The SIMVASCULAR platform [15,16,17,18], an open-source CFD solver designed for cardiovascular applications, facilitated the numerical simulation of blood flow patterns and pressure distributions within the cardiovascular system. To ensure the fidelity of the simulations, invasive and ultrasound data were incorporated as input parameters, providing real-world physiological conditions. These data, combined with the patient-specific models and boundary conditions, allowed for a comprehensive analysis of the hemodynamic interactions between aortic stenosis and coronary lesions. All methods described in this study were carried out in accordance with the relevant guidelines and regulations for research involving human subjects and the use of human tissue samples.

### 2.1. Data Collection

**Figure 3:**
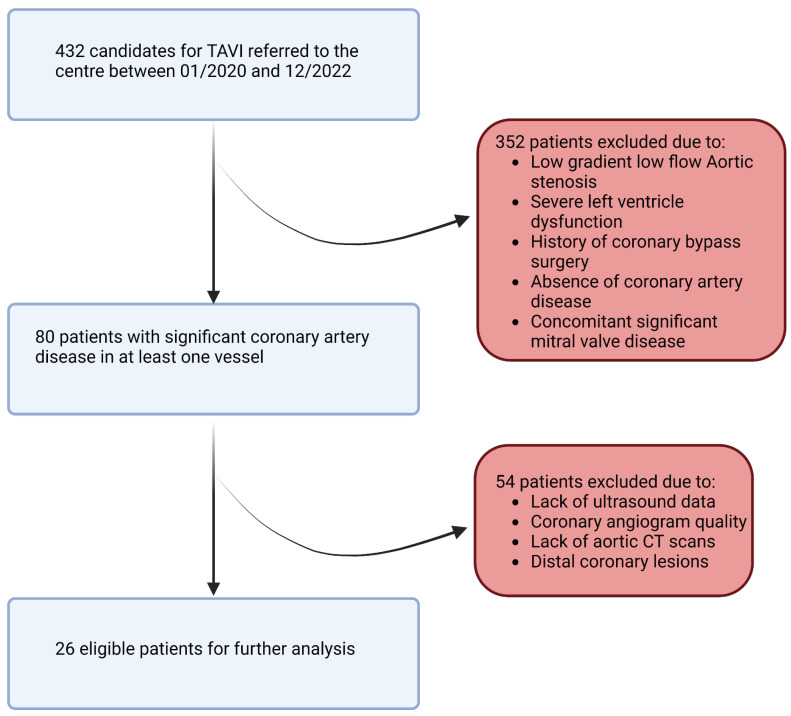
Study flowchart.

In this retrospective study, a population of 432 patients who underwent pre-TAVI coronary angiography between January 2020 and December 2022 was initially considered. After applying rigorous inclusion and exclusion criteria, a total of 26 patients and 26 stenosis (one per patient) were included in the final analysis. The inclusion criteria involved adult patients (age > 18 years) with symptomatic severe aortic stenosis and a confirmed indication for valve intervention. Patients with no history of coronary bypass surgery and those with documented coronary lesions showing stenosis (40–70%) in at least one of the proximal trunks were included. We note that patients with low-flow, low-gradient aortic stenosis and severe left ventricular dysfunction (left ventricular ejection fraction < 35%) were excluded from the study due to potential limitations in the accuracy of flow modeling (Figure 3). The informed consent of all patients and/or their legal guardian(s) was obtained as part of the FRANCE-TAVI registry enrollment policy; it includes the use of data for research related to the coronary artery disease and valvular heart disease. To sum up, a comprehensive dataset was assembled for each patient undergoing transcatheter aortic valve implantation (TAVI). Prior to the procedure, patients underwent retrospective cardiac CT, invasive coronary angiography, and cardiac ultrasound examinations to capture detailed anatomical and functional information along with invasive systolic, diastolic, and mean arterial and ventricular pressures to evaluate overall perfusion. FFR and instantaneous wave-free ratio (iFR) measurements were utilized to assess the functional significance of coronary lesions when available (all of them were performed before TAVI). After the procedure, additional cardiac ultrasound data were collected to assess post-procedural changes.

### 2.2. Data Preparation and 3D Model Creation

**Figure 4:**
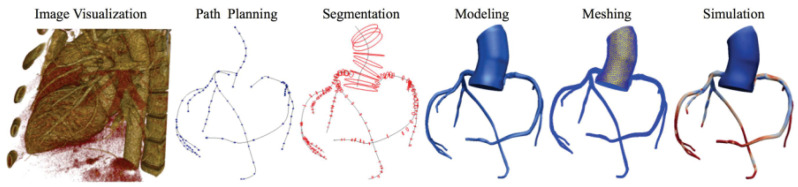
Simvascular platform pipeline for vascular simulations.

For each patient, the process of obtaining the 3D model involved meticulous manual segmentation based on both the aortic CT scan images and the coronary angiography data. Using the SIMVASCULAR workflow, the initial aorta was segmented by tracing its boundaries on each CT slice. Simultaneously, the major vessels, including the right coronary, left main, left anterior descending artery (LAD), and left circumflex (LCx), were designated by carefully identifying their positions. To ensure precision, the vessel diameters with each stenosis observed in the coronary angiography data were manually projected onto the segmented coronary centerlines. The resulting model underwent visual verification and necessary geometric filtering to obtain natural curves and enhance overall quality. Subsequently, for each patient, we obtained a 3D model consisting of the initial aorta and proximal segments, which was exported to the SLICER 3D software (v5.0.1), where a solid model was generated, containing the geometric details of the segmented structures. The generated product then underwent meshing using the TetGen plugin in SIMVASCULAR [19], resulting in a discretized representation with elements sized between 0.01 and 0.03 mm, depending on the required level of detail. A mesh independence test was performed by refining the mesh resolution (1.5 to 3.3 million elements) and assessing its impact on FFR, iFR, and WSS. The final resolution was chosen based on convergence criteria, ensuring changes in hemodynamic indices remained below 2% with further refinement. This comprehensive process ensured precise and accurate representation of the aorta and coronary arteries, enabling further analysis and simulations in subsequent stages of the study (Figure 4).

### 2.3. Boundary Conditions and Physiology Models

**Figure 5:**
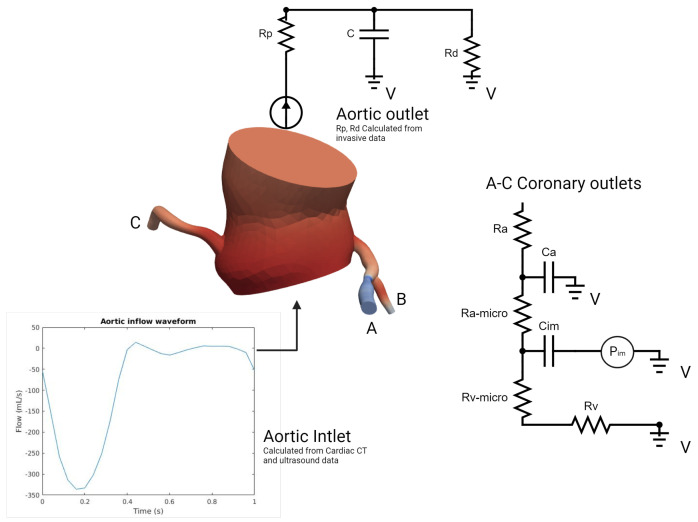
Problem specification of the inlet, ascending thoracic aorta, and coronary outlets for simulations of blood flow (Details in text).

In our model (Figure 5), which was inspired by the work of Mantero et al. [20,21], the systemic aortic outlet was represented by a classic Windkessel RCR boundary system, and consisted of three elements: a proximal resistance representing the viscous resistance of the arterial vasculature just downstream of the model ($R_{p}$), a capacitor modeling the compliance of all downstream vasculature (C), and a distal resistance representing the resistance of the capillaries and venous circulation ($R_{d}$). Tuning these parameters to accurately reflect patient-specific physiological conditions required iterative adjustments after first-order simulations until outputs closely aligned with the invasive aortic pressure curves for each patient (Table 1). Initially, the total resistance was calculated by dividing the mean pressure by the cardiac output, with a typical ratio of 7–11% used to distribute this resistance between Rp and Rd, considering that most vascular resistance is concentrated in the downstream vasculature, which was compatible with the data found in [22]. Compliance, which modulates the pressure waveform amplitude, was estimated by analyzing the diastolic behavior and the systolic–diastolic pressure difference. Once these parameters were determined, they were used as input for the simulation platform to apply the RCR boundary condition. In our case, this process was run for each patient in two different conditions: Before and immediately after valve replacement. Aortic inflow curves used for inlet input were obtained through contrast-phase cardiac magnetic resonance (PC-CMR) of two subjects with aortic stenosis acquired in our center with proper encoding velocities. Tuning flow curves for each patient was performed according to the aortic valve area and stroke volume obtained through cardiac CT left ventricle volumetry and Doppler imaging.

On the other hand, the coronary outlet was based on a modified Windkessel (Figure 5) and encompassed small artery resistance ($R_{a}$), microcirculation resistance ($R_{a\;\mathrm{micro}}$), venous resistance ($R_{v}$), arterial compliance ($C_{a}$), and intramyocardial compliance and pressure ($C_{\mathrm{im}}$, $P_{\mathrm{im}}$) [23]. The distribution of blood flow to each outlet was determined using a generalized form of Murray’s law [24]. At rest coronary blood flow was assumed constant, representing 4% of the cardiac output (CO) [20,25]. In order to simulate pharmacological stress under adenosine infusion at 140 mcg/kg/min, the pressure–flow relationship was modeled individually for each outlet using Murray’s law to capture locally induced pressure/flow drops due to stenosis [26,27,28,29,30], employing an iterative first-order simulations tuning process of 4–6 simulations (Table 2). Tuning the boundary condition parameters aimed to reflect the typical behavior of the epicardial pressure where no significant pressure drop is observed at rest but there is a more pronounced effect under stress conditions with elevated flow [26,27,28,29,30]. Briefly, total coronary resistances were computed based on the mean coronary flow, derived from cardiac output (CO) and projected mean pressures proportionate to the stenosis degree. Subsequently, resistances were distributed for the three parameters of Ra:Rm:Rv, with ratios of 0.32:0.52:0.16 at rest and 0.41:0.28:0.31 during stress as described in [20,31]. Total compliance of the right and left coronary arteries was obtained from the literature and distributed according to the respective section areas [20]. Left intramyocardial pressures were obtained through invasive ventricular pressures acquired at the time of TAVI procedure (before and after); the same $P_{\mathrm{im}}$ pressures were used in pharmacological stress conditions. To consider the physiological specificity of the right coronary artery and right ventricular pressure, a physiological right ventricle pressure waveform was obtained from a public repository [32] and tuned using Doppler data (tricuspid regurgitation velocity) [33] in order to respect the described coronary velocities ratio of 3:1 (left versus right) [34]. All our patients had right-dominant coronary arteries. Finally, total arterial compliance ($C_{a\;\mathrm{total}}$), defined as the sum of all coronary arterial compliances ($C_{a}$) and intramyocardial compliance ($C_{\mathrm{im}}$), was obtained from the literature and distributed among the outlets based on their cross-sectional areas, in respect to Murray’s law [20]. An iterative tuning procedure through first-order simulations was employed to determine the values of $C_{a\;\mathrm{total}}$ and $C_{\mathrm{im}\;\mathrm{total}}$ that produced the physiological ostial coronary pressure curves consistent with the invasive data for each patient.

#### 2.3.1. Fluid–Solid Models

For the fluid–solid models, the density of blood ρ was set to 1.06 kg/m^3^, and the viscosity μ was set to 0.04 P. As previously mentioned, reference values were assumed for the stiffness and wall thickness of the major vascular segments, including the ascending aorta and coronary arteries [20]. The coupling between the wall parameters and the three-dimensional vascular model was based on the work of Figueroa et al. [35].

#### 2.3.2. Equations Solution

The conservation equations for mass and linear momentum (Navier–Stokes equations) were solved for the flow of an incompressible Newtonian fluid in a deformable domain using the finite element analysis implemented in the open-source svSOLVER library within the SIMVASCULAR platform [36,37]. The calculations were performed on the Simvascular Super Computing Gateway [38] utilizing 96 cores with 64 GB of memory each. The solver parameters included a step Construction of 5 and maximum number of iterations for the svLS NS Solver of 10, which are linear solver settings that are optimized for coronary simulations, and achieved an average residual of 0.005. The simulations were run for 7 cardiac cycles until a cyclic pattern of pressure was achieved from one cycle to another, typically requiring 1–2 days of computation time. All reported results correspond to the 7th cycle.

#### 2.3.3. Vessel Wall Properties

Due to the current limitations of experimental data on variations in the mechanical properties and vessel thickness, uniform wall properties were assigned to each of the four sections of the vascular system. These properties were represented by the effective constant stiffness and wall thickness. The stiffness of the thoracic aorta was chosen to provide physiological levels of stress over the physiological range of blood pressure [39], while the modulus for the coronary vessels was based directly on experimental data [40]. The vessel wall thickness in each section was assumed to be 1/10th of the mean radius [41].

The simulation results were visualized using ParaView (Kitware, Clifton Park, NY, USA), which also facilitated post-processing.

#### 2.3.4. Calculation of Indices

The coronary flow reserve (CFR) is a measure of the ability of the coronary arteries to increase blood flow to the myocardium in response to increased demand. It is defined as the ratio of the maximal or hyperemic flow down a coronary vessel to the resting flow. CFR can be measured invasively with a Doppler-tipped coronary guidewire or by using a wire-based thermodilution technique. A normal CFR is considered to be greater than 2.0 and in most patients should be somewhere between 3 and 5. However, there is a range of normal CFR values between approximately 2.5 and 6, so a value of 3.0 could be normal in one patient and abnormal in another [42].

The equation for the calculation of *CFR* is as follows:

$$CFR=\frac{max\;\mathrm{blood}\;\mathrm{flow}}{resting\;\mathrm{blood}\;\mathrm{flow}}$$

Invasively measured CFR has largely been abandoned as a method for interrogating intermediate coronary lesions because of its limitations [43]. However, it can be used to assess microvascular function in patients with normal-appearing epicardial coronary vessels.

The fractional flow reserve (FFR) is a measure of the functional significance of a coronary artery stenosis. It is calculated as the ratio of the pressure distal to the stenosis (Pd) to the pressure proximal to the stenosis (Pa). A normal FFR is 0.80 or greater, which means that the coronary artery is able to increase blood flow to the heart muscle adequately in response to increased demand [44].

FFR is measured during cardiac catheterization, a minimally invasive procedure in which a catheter is inserted into a coronary artery in the groin or arm and then threaded to the heart. Once the catheter is in place, a pressure sensor is advanced to the site of the stenosis. The pressure sensor measures the pressure distal to the stenosis, and the pressure proximal to the stenosis is measured directly from the coronary artery. The *FFR* is then calculated using the following equation:

$$FFR=\frac{Pd}{Pa}$$

where: *Pd* = pressure distal to the stenosis, *Pa* = pressure proximal to the stenosis

An *FFR* of less than 0.80 indicates that the stenosis is hemodynamically significant and causing a decrease in blood flow to the cardiac muscle. In these cases, there is a risk of myocardial ischemia [6], which is a condition in which the heart muscle does not receive enough oxygen. FFR is a valuable tool for assessing the functional significance of coronary artery stenoses. It is more accurate than other methods, such as coronary angiography, for predicting which patients are at risk of myocardial ischemia [45]. FFR is also used to guide treatment decisions, such as whether to perform coronary angioplasty or bypass surgery [46].

The instantaneous wave-free ratio (iFR) is a newer physiologic measurement that utilizes similar principles to FFR but does not require the use of a hyperemic agent. In the iFR-SWEDEHEART trial, iFR was non-inferior to FFR in the prediction of myocardial ischemia [47]. A network meta-analysis further justified the use of iFR, revealing that iFR-guided revascularization was non-inferior to FFR-guided revascularization for major adverse cardiac events at 1-year follow-up [48].

In iFR, the same pressure wires utilized in FFR are passed to a point distal to a stenotic lesion. During a period of diastole known as the “wave-free period”, iFR then calculates the ratio of the distal coronary artery pressure (Pd) to the pressure within the aortic outflow tract (Pa). During this time-frame, interactions of blood flow complicating these measurements are negligible. Lesions found to have a Pd/Pa ratio less than 0.89, are determined to be significant and have been shown to be non-inferior to the FFR cutoff of 0.8 [45].

Coronary artery lesions determined to have iFR ratios less than 0.89 and FFR ratios less than 0.8 currently are recommended to undergo further treatment with PCI [6]. As it is still a newer technology, some providers consider an iFR ratio of 0.86 to 0.93 to be an area of uncertainty and recommend a hybrid approach utilizing evaluation with FFR [49].

### 2.4. Statistical Analysis

**Figure 6:**
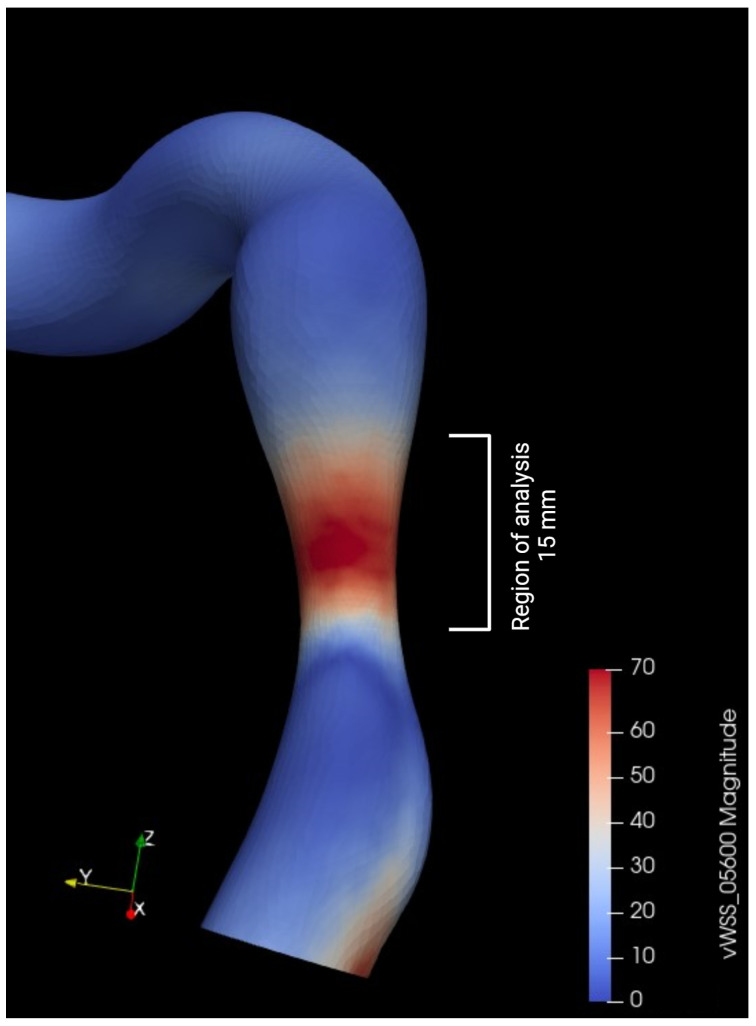
Metrics used for wall shear stress (WSS) analysis.

**Figure 7:**
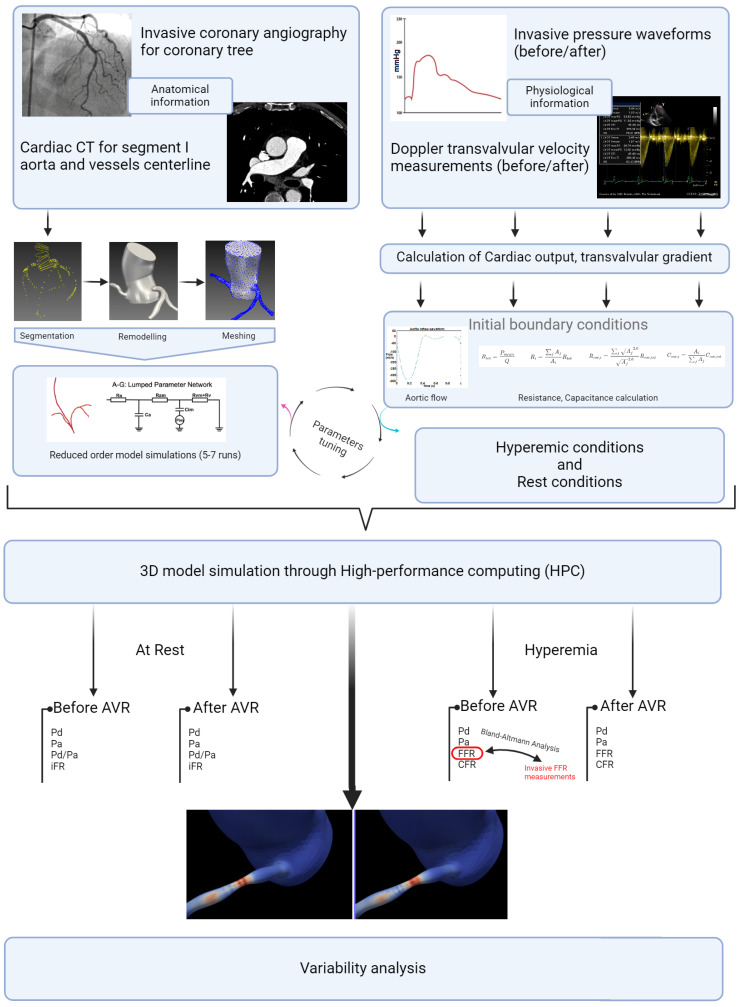
Study detailed workflow.

Descriptive statistical analysis focused on the epidemiological characteristics of the population, the degrees of stenosis, and the length measured by QCA. A *t*-test (the paired *t*-test or the Wilcoxon signed-rank test, depending on the distribution) was used to assess differences in hemodynamics before and after valve intervention. For the external validation of our FFR estimation method, a Bland–Altman analysis was conducted to examine the agreement with invasive values (in 10 patients). For the analysis of hyperemic and non-hyperemic parameters, as well as shear stress before and after intervention, a simple *t*-test (the paired *t*-test or the Wilcoxon signed-rank test, depending on the distribution) was also performed. The parameters used for shear stress analysis included the time average shear stress in these areas (TAWSS) and the Oscillatory Shear Index (OSI) (Figure 6). The significance threshold was set below 0.05. The central figure (Figure 7) illustrates all the steps described in this section, as well as the progression of the statistical analysis. The protocols conducted in this study were approved by the ethics committee of Brest university hospital.

## 3. Results

### 3.1. Study Population

The analysis included 26 patients with a mean age of 79.6 years. Hypertension (92%) was the most predominant cardiovascular risk factor, followed by dyslipidemia, diabetes, and smoking (77%, 31%, and 23%, respectively). The overall left ventricular ejection fraction (LVEF) was preserved, with a median of 58.5% (6.6%), and the presence of left ventricular hypertrophy (96%) was nearly constant. Significant mitral valve abnormalities were documented in 15% of cases (Table 3). Angiographically, the left anterior descending artery (LAD) was the most affected (62%), followed by the right coronary artery (RCA), left circumflex (LCx), and ramus intermedius (23%, 12%, and 3.8%, respectively). Quantification of stenosis degree by QCA revealed a median of 56% (with a range from 40% to 71%), with stenosis length ranging from 8 mm to 23 mm (mean and median of 15 mm) (Table 4).

### 3.2. Hemodynamic Conditions Before and After Valve Intervention

Analysis of hemodynamic variables before and after valve intervention showed no significant difference in systolic, diastolic, and mean pressures individually (*p* = 0.11, 0.72 and 0.14, respectively). Furthermore, a significant increase in stroke volume (79.1 vs. 81.4 mL, *p* < 0.01) and aortic valve area (0.6 vs. 2.4 ${\mathrm{cm}}^{2}$, *p* < 0.01) was observed, along with a marked decrease in systolic ejection time (336 vs. 252 ms, *p* < 0.001) (Table 5).

### 3.3. Validation of CFD Method vs. INVASIVE Measurement

**Figure 8:**
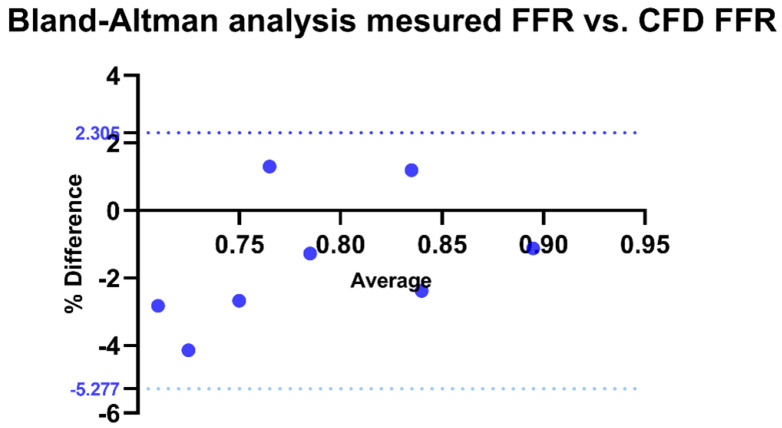
Bland–Altman analysis of invasive FFR vs. CFD-derived FFR. Agreement between invasive and CFD-derived FFR, with the x-axis representing their average and the y-axis showing the percentage difference. The solid black line indicates the mean bias, while the dotted blue lines represent the Limits of Agreement (LOA: −5.277% to 2.305%). CFD: Computational fluid dynamics.

For the validation of our method, a Bland–Altman analysis was conducted to compare the correlation between FFR calculated by CFD and invasively measured FFR. The bias was estimated at −1.486 with a standard deviation of 1.938. All measurements fell within the limits of agreement, with 100% agreement rate (*p* < 0.0001) (Figure 8).

### 3.4. Variation in Non-Hyperemic Indices

**Figure 9:**
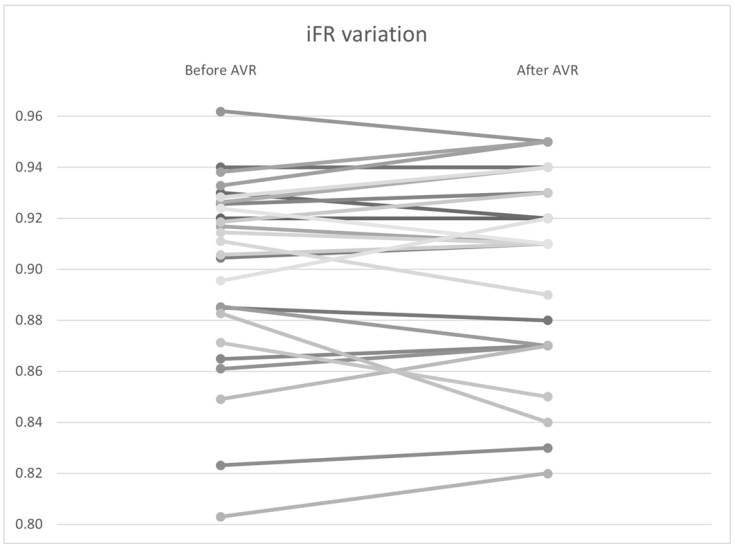
*iFR* variation by patient before and after AVR. AVR: Aortic valve replacement.

To investigate the variation in non–hyperemic indices, including the Pd/Pa ratio, iFR, and total coronary flow at rest, a Wilcoxon signed–rank analysis was performed. No significant differences were found in the pressure variables, including Pd/Pa (0.940 vs. 0.942, *p* = 0.94) and iFR (0.901 vs. 0.901, *p* = 0.67) (Figure 9). However, there was statistically significant variability in coronary flow, with a flow increase from 4.95 to 5.13 mL/s (*p* < 0.01) (Table 6).

### 3.5. Variation in Hyperemic Indices

**Figure 10:**
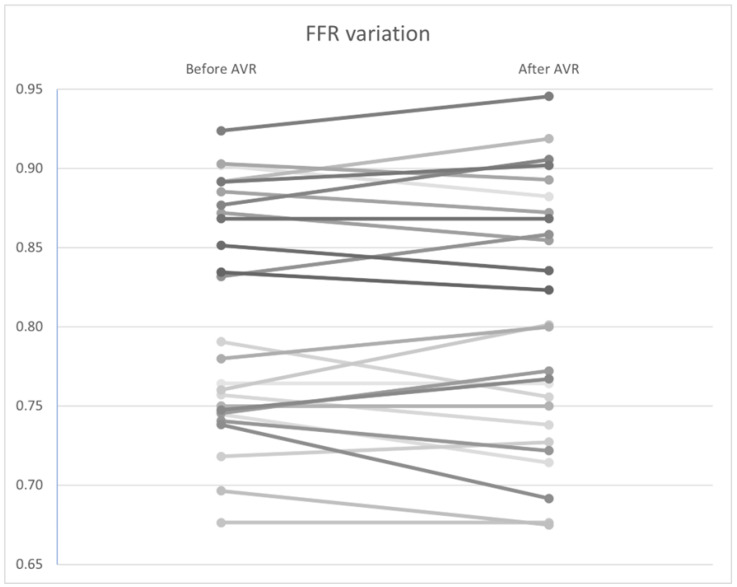
*FFR* variation by patient before and after AVR. AVR: Aortic valve replacement.

**Figure 11:**
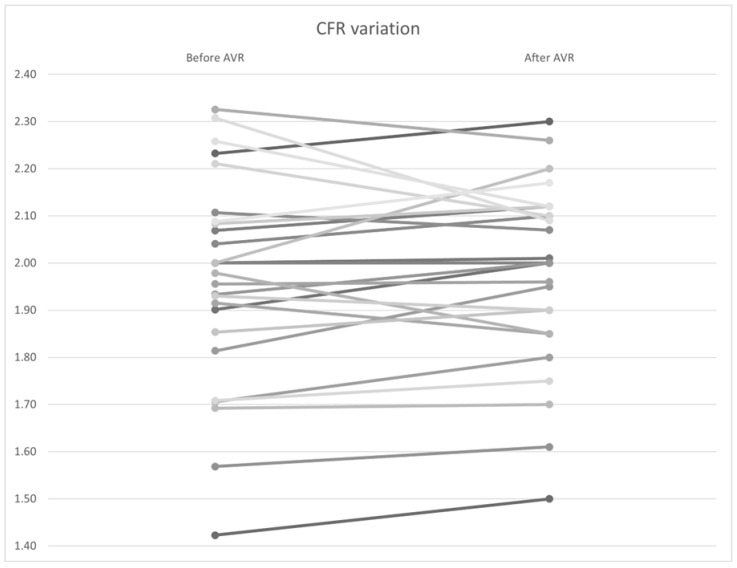
*CFR* variation by patient before and after AVR. AVR: Aortic valve replacement.

Similar to the non–hyperemic indices, no statistically significant differences were found in FFR (Figure 10), CFR (Figure 11), and Pa and Pd pressures (*p* = 0.92, 0.34, and 0.98, and 0.97, respectively). However, a significant improvement in hyperemic coronary flow was observed, with an increase from 9.71 to 10.2 mL/s (*p* < 0.01) (Table 7).

### 3.6. Variation in Shear Stress Parameters

**Figure 12:**
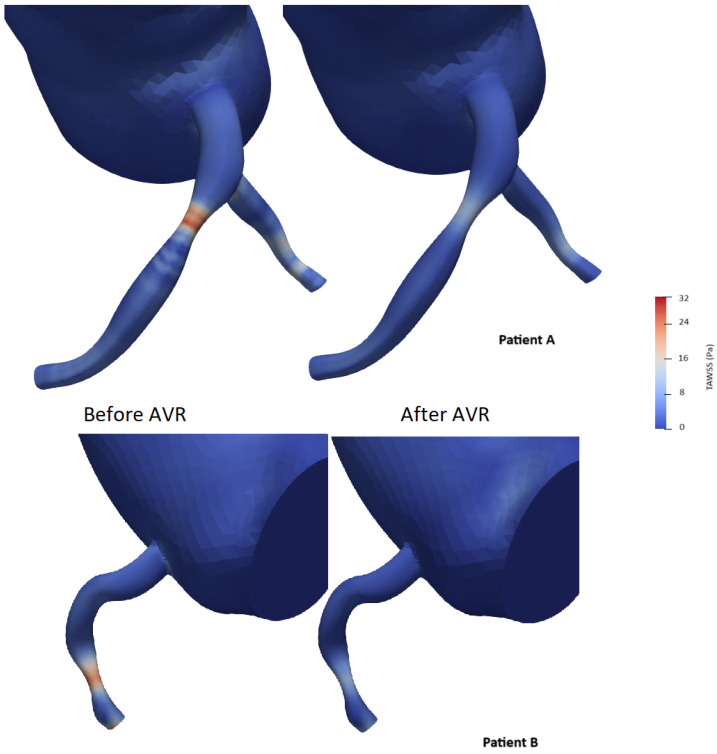
Examples of time average wall shear stress distribution (TAWSS) (in pascal) in two patients before and after aortic valve replacement (AVR).

To simplify the shear stress analysis, we focused on the segment 15 mm upstream of the lesion and conducted a statistical analysis by selecting areas on the 3D model and retrieving time average shear stress values (TAWSS). The mean and median shear stress values were found to be lower after valve intervention (21.0 vs. 17.5 Pa and 22.4 vs. 17.4 Pa with *p* < 0.05, respectively) (Table 8). The area exposed to high shear stress (defined as the percentage of elements exposed to values exceeding four times the TAWSS on the segment) varied significantly, showing a significant decrease after valve intervention (59% vs. 40.8%, *p* < 0.001) (Figure 12). The Oscillatory Shear Index (OSI) was calculated to evaluate the degree of flow reversal before and after transcatheter aortic valve implantation (TAVI). OSI values showed a significant reduction from 0.32 before TAVI to 0.21 post-TAVI (*p* < 0.001), indicating a decrease in oscillatory shear stress and improved hemodynamic stability following the procedure.

## 4. Discussion

Consistent with the literature, the prevalence of coronary artery disease in our cohort reflects its association with age. The constant presence of hypertension is indicative of its association with the genesis of aortic disease [50]. The prevalence of other cardiovascular risk factors reflects the interaction between coronary artery disease and degenerative aortic stenosis. The frequent presence of left ventricular hypertrophy (LVH) can be explained by hypertension and the increased afterload caused by severe aortic stenosis. The left anterior descending artery remains the most affected artery in patients with aortic stenosis, as reported in several previous studies [51,52,53,54]; no mechanistic explanation could be found for this finding.

The improvement in the functional aortic surface area and ejection time documented through our study is consistent with different studies [40,55], which can be attributed to a better opening profile of the prosthesis and elimination of systolic obstruction. However, we did not note any immediate improvement in the pressure parameters, systolic ejection volume, nor ejection fraction, which was previously reported [56]. Seppelt et al. observed a reduction in myocardial contractility markers immediately after transcatheter aortic valve implantation (TAVI). Cardiac work at the same pre-load and end-systolic elastance significantly decreased compared to baseline. In addition to reduced contractility, the same team observed impaired diastolic function shortly after valve implantation. The time constant of relaxation (Tau), an independent measure of isovolumetric relaxation pre-load, and dP/dtmin increased compared to baseline [57]. As a result, both the diastolic pressure and end-systolic volume increased after valve implantation. McConkey et al. suggest that patients with aortic stenosis and preserved left ventricular ejection fraction (LVEF) with left ventricular hypertrophy have decreased contractile reserve (as illustrated in our cohort by CFR measurements), especially during increased heart rate [58]. However, all these findings are based on small cohort studies, awaiting randomized controlled trials for formal conclusions.

Our method used patient-specific hemodynamic and imaging data unlike most commercial solutions which use imaging data exclusively. Our analysis revealed excellent agreement between invasive FFR values and those calculated by computational fluid dynamics (CFD) through the Bland–Altmann analysis we performed. Several studies have confirmed this agreement for CFD-based techniques (such as FFRct, vFFR, and QFR), with excellent results. The feasibility and diagnostic performance of FFRct were evaluated in the DISCOVER FLOW trial [59]. This prospective study analyzed 103 patients (159 lesions) who underwent coronary computed tomography angiography (CTA) and invasive angiography with FFR measurements. The correlation between CFD-based FFR and invasive FFR values was very good, with improved performance compared to CTA alone. FFRct in this study achieved a sensitivity of 88% and a negative predictive value (NPV) of 92%, with higher specificity (82%) and positive predictive value (PPV) of 74%, leading to an overall improvement in diagnostic accuracy of 25% [59]. Tanigaki et al. reported that QFR obtained from coronary angiography showed strong correlation with invasive FFR (r = 0.77 to 0.85) and high diagnostic performance for predicting FFR < 0.81 (accuracy of 85% to 92%, sensitivity of 74% to 94%, and specificity of 91% to 93%) [60]. In a sub-analysis of the NXT trial, in stable patients undergoing coronary angiography, an FFR CT value < 0.81 was a predictor of long-term cardiovascular events leading to planned and unplanned revascularizations. This was superior to the presence of significant stenoses on CTA alone. Furthermore, the FFR CT value was an independent predictor of outcomes [9]. This opens the door to more studies on the prognostic value of these methods, such as the FAVOR III trial, which concluded with the superiority of QFR over angiography-guided revascularization alone for a composite primary endpoint (rate of major adverse cardiac events at 1 year, including all-cause death, myocardial infarction, or ischemia-driven revascularization) [61]. Further studies are needed to highlight the clinical and cost-effectiveness benefits.

One of our major findings was the insignificant variability of non-hyperemic indices before and after valve interventions, which has been reported in several studies. In a single-center study by R. Scarsini et al., the iFR value did not change significantly after TAVI (iFR pre-TAVI 0.88 [0.85–0.96], iFR post-TAVI 0.90 [0.83–0.93]; *p* = 0.30), with good agreement between measurements in the Bland–Altman analysis [56]. However, iFR values were below the conventional ischemic threshold of 0.89 in 47.8% of patients before TAVI and 26.1% after (McNemar’s test *p* = 0.22). Thus, there is no valid threshold after TAVI. Despite the advantage of avoiding hyperemic agent administration, underestimation/overestimation of the functional significance of the lesion is possible for any initial iFR value, leading to a higher rate of lesion reclassification during long-term follow-up compared to FFR. Nevertheless, this appears to be related to the distribution of pre-TAVI iFR values clustering around the 0.89 threshold rather than inherent variability [56].

In the same direction, our study found no significant variability in hyperemic indices. Data on the variability of hyperemic indices in the literature are inconclusive. Pesarini et al., in a prospective single-center study, observed a reclassification rate of lesion significance of 6–7% of cases (8/133 patients), despite an overall non-significant difference in FFR values [55]. As such, questions have arisen regarding the safety of an FFR-guided revascularization strategy in patients with aortic stenosis. This was investigated by Benseba et al., who observed no significant difference in major adverse cardiovascular and cerebrovascular events (MACCE) between the angiography-guided (42.4%) and FFR-guided (37.4%) groups during a mean follow-up of 33.7 months (*p* = 0.333). When comparing the results of the FFR-guided PCI group (32.7%) with the PCI-guided angiography group (46.4%), no significant difference was observed (*p* = 0.999). Only one adverse event occurred after intracoronary adenosine administration [62]. Based on these data, and despite the lack of prognostic benefit, the safety of the technique in this population is validated. However, randomized controlled trials are yet to confirm these results.

One of our interesting findings is the overall reduction in the coronary flow reserve, which reflects a certain level of microvascular dysfunction, with two main hypotheses proposed to explain that (Figure 1). The first hypothesis suggests that microvascular dysfunction leads to myocardial ischemia, as initially proposed by Ahn et al., who demonstrated reduced myocardial perfusion reserve in patients with aortic stenosis using cardiac perfusion magnetic resonance imaging [63]. The second hypothesis is that signs and symptoms of ischemia result from high wall stress and mechanical effects in response to aortic stenosis (including increased arteriolar vasodilation and wall stress) [64]. The lack of immediate improvement in CFR in our study was not surprising given similar findings in previous studies. Doty et al. examined intraoperative CFR during aortic valve replacement and found that CFR did not immediately increase despite a reduction in transvalvular gradient [65]. They concluded that the regression of left ventricular hypertrophy was necessary for CFR improvement. The relationship between CFR improvement and regression of LVH was supported by the work of Eberli et al. [66].

To our knowledge, no study has investigated the interaction between proximal coronary shear stress and aortic stenosis. Several studies have highlighted the beneficial effect of aortic valve replacement on aortic shear stress without including the coronary tree in the analysis [67,68,69]. Studies based on intravascular imaging have associated both low and high wall shear stresses with aspects of plaque progression and vulnerability, but the precise relationships remain uncertain, and the findings are sometimes controversial. Low wall shear stress (WSS) has been linked to endothelial dysfunction and plaque progression, increasing the risk of future angiography-guided revascularizations and major adverse cardiovascular events. Conversely, high WSS—defined as regions exceeding four times the mean regional TAWSS—has been associated with excessive mechanical stress on the endothelium, potentially contributing to plaque rupture and future myocardial infarction. Additionally, elevated Oscillatory Shear Index (OSI), which reflects disturbed and bidirectional flow, has been correlated with atherosclerosis-prone regions and adverse coronary outcomes [70,71]. In our study, we observed a clear reduction in the vascular surface exposed to major shear forces. This effect does not seem to be solely the result of restoring “normal” hemodynamics (Figure 12).

## 5. Limitations

Our study on the interaction between coronary circulation and severe aortic stenosis represents a novel contribution to the literature, but this work is subject to several notable limitations that warrant consideration.

Firstly, we did not incorporate the dynamic motion of the heart during the cardiac cycle into our computational model. This omission may introduce inaccuracies in our findings, as the movement of the heart plays a crucial role in shaping coronary hemodynamics.

Secondly, while we successfully replicated the downstream epicardial arteries in our lumped parameter boundary condition, the exclusion of the location of coronary outlets and a segment of the epicardial vessels from our three-dimensional model is a notable limitation. This exclusion may impact the fidelity of our simulations, potentially leading to incomplete representations of coronary flow dynamics.

Thirdly, our assumption of a uniform Young’s modulus across the entire computational model overlooks the spatial variability in vessel wall properties. This oversimplification may compromise the precision of our results. Future research efforts should focus on developing noninvasive techniques for accurately estimating wall thickness and elastic properties to address this limitation. On the other hand, shear stress parameters are highly sensitive to parameter tuning and geometrical modeling, which we tried to avoid through a statistical approach; thus, caution is advised and no reliable conclusions can be drawn from this work. Moreover, our reliance on rigid vessel walls represents a simplifying assumption that may not fully capture the compliant nature of vascular structures. While fluid–structure interaction simulations have been explored by other research groups [72,73], the construction of a robust, compliant 3D model surrogate for tuning remains a challenge that requires further investigation in subsequent studies.

Furthermore, the modeling of the coronary tree was based on coronary angiography, which, despite its good spatial resolution (250 µm), provides only two-dimensional projections. Intracoronary imaging represents the optimal tool but remains inaccessible for frequent use. Lastly, the sample size used in this study limits the statistical power for robust conclusions but can serve as a foundation for future studies.

## 6. Conclusions

This study highlights the usefulness of computational fluid dynamics (CFD) in cardiovascular physiology, demonstrating its accuracy in assessing coronary hemodynamics and validating its agreement with invasive measurements. Despite the hemodynamic changes induced by transcatheter aortic valve implantation (TAVI), key physiological indices (FFR, iFR, and CFR) remained stable, which may confirm their reliability in guiding revascularization decisions. Additionally, TAVI led to a significant reduction in coronary shear stress and flow oscillations, suggesting a potential protective effect on plaque stability. However, further studies are needed to validate these findings and assess their long-term clinical implications.

## Figures and Tables

**Table 1:** Parameter tuning and validation details for aortic outlets.

Aortic Outlet	Value	Tuning Process	Validation Parameters
Rp	90% of total resistance	First-order simulations with 0.5% steps	Systolic and diastolic aortic pressure curves
Rd	10% of total resistance	First-order simulations with 0.5% steps	Systolic and diastolic aortic pressure curves
C	Initial value 0.001 cm^5^/dyne	First-order simulations with 0.0002 cm^5^/dyne steps	Systolic and diastolic aortic pressure curves

**Table 2:** Parameter tuning and validation details for coronary outlets.

Coronary Parameter	Value	Tuning Process	Validation Parameters
Total coronary flow	4% of cardiac outflow at rest, 10–12% in stress conditions	In stress, flow adapted to stenosis degree in each coronary artery with 0.05 mL/s steps for first-order simulations	Ostium and distal coronary pressures
Total coronary resistance	Calculation using coronary flow and pressure	First-order simulations with 100 dynes/cm^2^ steps	Ostium and distal coronary pressures
Pim	Left ventricle invasive pressures (during TAVI)	No tuning needed	Ostium and distal coronary pressures
Rvmic	Supposed 0	No tuning needed	Ostium and distal coronary pressures
Ra	32% of total coronary resistance at rest, 41% in stress	First-order simulations with 3% steps	Ostium and distal coronary pressures
Ramic	52% of total coronary resistance at rest, 28% in stress	First-order simulations with 3% steps	Ostium and distal coronary pressures
Rv	16% of total coronary resistance at rest, 31% in stress	First-order simulations with 3% steps	Ostium and distal coronary pressures
Ccor	Initial value of $3.6\times 10^{-5}$ cm^5^/dyne for left coronary arteries and $2.5\times 10^{-5}$ cm^5^/dyne for right	First-order simulations with $0.05\times 10^{-5}$ cm^5^/dyne steps	Ostium and distal coronary pressures
Cim	89% of total coronary compliance	First-order simulations with 1% steps	Ostium and distal coronary pressures
Ca	11% of total coronary compliance	First-order simulations with 1% steps	Ostium and distal coronary pressures

**Table 3:** Study population characteristics, LAD: Left anterior descending, RCA: Right coronary artery, Lcx: Left circumflex, Ram inter: Ramus medium, CKD: Chronic kidney disease, HTN: Hypertension, CAD: Coronary artery disease, BMI: Body mass index, LVEF: Left ventricle ejection fraction, QCA: Quantitative coronary angiography.

Parameter	%
Artery LAD Disease	16 (62%)
Artery RCA Disease	6 (23%)
Artery LCx Disease	3 (12%)
Artery Ram Inter Disease	1 (3.8%)
CKD (Chronic Kidney Disease)	4 (15%)
Diabetes	6 (23%)
Dyslipidemia	20 (77%)
HTN (Hypertension)	24 (92%)
Hx of CAD (History of Coronary Artery Disease)	4 (15%)
LVH (Left Ventricular Hypertrophy)	25 (96%)
Mitral VD (Mitral Valve Disease)	4 (15%)
Smoking	8 (31%)

**Table 4:** Study population characteristics, LAD: Left anterior descending, RCA: Right coronary artery, Lcx: Left circumflex, Ram inter: Ramus medium, CKD: Chronic kidney disease, HTN: Hypertension, CAD: Coronary artery disease, BMI: body mass index, LVEF: Left ventricle ejection fraction, QCA: Quantitative coronary angiography.

Parameter	N	Mean (SD)	Median [Q25–Q75]	Min	Max
Age (years)	26	79.6 (10.1)	82.0 [79.0; 85.0]	50.0	89.0
BMI (kg/m^2^)	26	25.7 (2.97)	25.4 [23.3; 28.5]	20.0	31.0
LVEF (%)	26	57.8 (6.60)	58.5 [55.0; 62.0]	45.0	70.0
Stenosis (QCA)	26	0.568 (0.0853)	0.580 [0.505; 0.637]	0.400	0.710
Length (mm)	26	15.1 (4.36)	15.0 [12.0; 18.0]	8.00	23.0

**Table 5:** Cardiac hemodynamic variation before and after AVR. AP: Arterial pressure, AVR: Aortic valve replacement, AVA: Aortic valve area.

Parameter	Before AVR (n = 26)	After AVR (n = 26)	ΔMean	*p*
Diastolic aortic pressure (mmHg), mean (SD)	78.5 (10.2)	78.3 (11.1)	−0.231	0.72
Mean aortic pressure (mmHg), median [Q25–Q75]	99.0 [92.7; 108]	99.8 [92.3; 107]	0.526	0.14
Systolic aortic pressure (mmHg), mean (SD)	141 (19.2)	143 (20.8)	2.04	0.11
Stroke volume (mL), mean (SD)	79.1 (17.0)	81.4 (16.4)	2.31	<0.01
Ejection time (ms), mean (SD)	336 (27.3)	252 (20.4)	−83.9	<0.001
AVA (cm^2^), median [Q25–Q75]	0.600 [0.542; 0.700]	2.40 [2.17; 2.80]	1.86	<0.001

**Table 6:** Non–hyperemic indexes variation before and after AVR. Pa: Proximal pressure, Pd: Distal to stenosis pressure, iFR: Instantaneous wave-free ratio, Qrest: Coronary blood flow at rest.

Parameter	Before AVR	After AVR	ΔMean	*p*
Pa (mmHg), median [Q25–75]	95.7 [89.8; 105]	95.8 [88.8; 106]	0.0042	0.96
Pd (mmHg), median [Q25–75]	90.0 [84.5; 99.0]	89.5 [84.5; 96.0]	−0.0962	0.91
Pd/Pa, median [Q25–75]	0.940 [0.920; 0.962]	0.942 [0.922; 0.949]	−0.00106	0.94
IFR, mean (SD)	0.901 (0.0376)	0.901 (0.0386)	0.000424	0.67
Qrest (mL/s), mean (SD)	4.95 (0.747)	5.13 (0.770)	0.185	<0.01

**Table 7:** Hyperemic indexes variation before and after AVR. Pa: Proximal pressure, Pd: Distal to stenosis pressure, FFR: Fractional flow reserve, CFR: Coronary flow reserve, Qhyper: Coronary blood flow at hyperemia.

Parameter	Before (n = 26)	After (n = 26)	ΔMean	*p*
Pa (mmHg), median [Q25–75]	99.7 [92.3; 107]	99.8 [92.5; 106]	0.004	0.98
Pd (mmHg), mean (SD)	80.5 (11.5)	80.4 (12.0)	−0.0962	0.97
FFR, mean (SD)	0.805 (0.0740)	0.804 (0.0807)	−0.00106	0.92
CFR, mean (SD)	1.97 (0.222)	1.98 (0.195)	0.0126	0.34
Qhyper (mL/s), mean (SD)	9.71 (1.83)	10.2 (2.00)	0.461	<0.01

**Table 8:** Wall shear stress parameters in region of interest before and after AVR vWSS: virtual wall shear stress.

Parameter	Before (n = 26)	After (n = 26)	ΔMean	*p*
Time average WSS (TAWSS) (Pa), median [Q25–75]	21 [13.71; 29.8]	17.5 [11.8; 27.0]	3.83	<0.01
Time average WSS (Pa), mean (standard deviation)	22.4 (10.85)	17.4 (9.77)	5.48	0.004
Percentage of elements exposed to high TAWSS, mean (standard deviation)	0.595 (0.02)	0.408 (0.181)	−0.187	<0.001
Oscillatory Shear Index (OSI), mean (standard deviation)	0.32 (0.02)	0.21 (0.03)	−0.04	<0.001

## Data Availability

The de-identified datasets generated and/or analyzed during the current study are not publicly available due to the hospital policies, but are available from the corresponding author on reasonable request.
